# Kinect V2 Performance Assessment in Daily-Life Gestures: Cohort Study on Healthy Subjects for a Reference Database for Automated Instrumental Evaluations on Neurological Patients

**DOI:** 10.1155/2017/8567084

**Published:** 2017-11-22

**Authors:** Alessandro Scano, Andrea Chiavenna, Matteo Malosio, Lorenzo Molinari Tosatti

**Affiliations:** Institute of Industrial Technologies and Automation (ITIA), Italian National Research Council (CNR), Via Corti 12, 20133 Milan, Italy

## Abstract

**Background:**

The increase of sanitary costs related to poststroke rehabilitation requires new sustainable and cost-effective strategies for promoting autonomous and dehospitalized motor training. In the Riprendo@Home and Future Home for Future Communities research projects, the promising approach of introducing low-cost technologies that promote home rehabilitation is exploited. In order to provide reliable evaluation of patients, a reference database of healthy people's performances is required and should consider variability related to healthy people performances.

**Methods:**

78 healthy subjects performed several repetitions of daily-life gestures, the reaching movement (RM) and hand-to-mouth (HtMM) movement with both the dominant and nondominant upper limbs. Movements were recorded with a Kinect V2. A synthetic biomechanical protocol based on kinematical, dynamical, and motor control parameters was used to assess motor performance of the healthy people. The investigation was conducted by clustering participants depending on their limb dominancy (right/left), gender (male/female), and age (young/middle/senior) as sources of variability.

**Results:**

Results showed that limb dominancy has minor relevance in affecting RM and HtMM; gender has relevance in affecting the HtMM; age has major effect in affecting RM and HtMM.

**Conclusions:**

An investigation of healthy subjects' upper limb performances during daily-life gestures was performed with the Kinect V2 sensor. Findings will be the basis for a database of normative data for neurological patients' motor evaluation.

## 1. Introduction

According to the World Health Organization (WHO) [1], “the essential components of successful neurorehabilitation include expert multidisciplinary assessment, goal-oriented programs and evaluation of impact on patient and goal achievement through the use of clinically appropriate, scientifically sound outcome measures incorporating the patient's perspective.” Consequently, the evaluation of motor performances of neurological patients is a standard practice in the clinical environment [2]. Evaluations that allow following the clinical course of the patient, orienting the therapies to administer, and measure their effect provide valuable assessment. Standard tools for the assessments are clinical scales. Clinical scales are surveys and questionnaires that associate a score to specific performances, assessing body function, activity, and participation issues according to the *International Classification of Functioning* (ICF) [3, 4]. Despite providing a wide variety of assessments, clinical scales are inter- and extraoperator dependent and have intrinsic low sensibility; furthermore, they suffer from ceiling and floor effects [5, 6].

In order to provide deeper and quantitative assessment, motion analysis is one of the main techniques used in clinics to assess the motor capabilities of neurological patients. The clinical status and the effects of the therapies can be evaluated in terms of restored motor performances, related, for example, to articular range of motion or quality of motor control [7, 8]. For such purposes, in clinical environments, marker-based optoelectronic systems are used [9–14]. They allow sampling the position of anatomical points of interest with high frequency and precision. While being the state-of-the-art devices for motion analysis, marker-based systems are expensive, require time-consuming acquisition and marker-positioning procedures, and are not likely to be adopted outside of the clinical environment. Furthermore, evidences that suggest that motor benefits obtained with rehabilitation are preserved only if the motor function is kept under training are growing, possibly if they are specifically task-oriented and with high intensity [15]. Thus, rehabilitation and consequent motor monitoring and evaluation are seen as a never-ending process that should be carried on also in the home environment and daily life in order to maintain the functional improvements obtained in the clinics. Furthermore, rehabilitation can provide positive outcome only with regular use of the functionality under training. In fact, according to the Schema Theory of Motor Learning [16], valid for the description of both physiology and pathology, brain-stored dynamical models underlying movement (motor programs) are better mastered if they are continuously trained. Even more importantly, sanitary costs associated to clinical neurological rehabilitation and evaluation exams are growing consistently [17, 18]. Following these premises, instruments capable of supervising rehabilitation and home training acquire valuable and strategic potential and, in particular, the ones that allow motion tracking.

As deeply described in [17], two possible training approaches can be identified for home training: coaching and gaming, each one featuring its own advantages. The *coaching* method relies on intensity of repetition of specific motor gestures and biomechanical analysis and evaluation of the quality of motion, while *gaming*-based training enhances motivation and participation, probably guaranteeing more training regularity in time. In particular, the coaching approach, which will be investigated in this study, is more suitable for intensive training and especially for providing standardized motor evaluation.

Many portable and relatively cheap devices that allow the tracking of human movements, usable in the domestic environment, can be associated to coaching or gaming rehabilitation and evaluation strategies. Among them, Microsoft Kinect and Kinect V2, Asus Xtion, XSens, Intel Creative, Leap Motion, Nintendo Wii Balance Board, and Wiimote are technologies that can be used to track human motion. Being low cost, widely supported, and featuring human tracking, resembling the ones of marker-based systems, the Microsoft Kinect sensor found wide application in the medical and rehabilitation fields. An important difference must be underlined: marker-based systems do not measure directly joint centers, while Kinect-embedded algorithms produce an estimation of joint centers based on image segmentation and depth. The first generation of the Microsoft Kinect sensor was released in 2010 for videogame industry. Its embedded algorithms allow the tracking of 20 human joint kinematics. Kinect versatility was exploited in medical and rehabilitative applications, including assistance to motor gestures to children or neurological patients in general [19, 20], evaluation of cognition during training [21], training of selective movements of the pelvis [22], and supporting rehabilitative sessions during the execution of functional motor tasks connected to virtual reality environments [23–27]. Other works evaluated motor performances [28, 29], confirming coherency with results obtained with marker-based systems and clinical scale evaluations. A review by [30] comes to the conclusion that Kinect can be considered as an adequate tool for supporting rehabilitation; a review [31] concludes that expected future works with the Kinect for rehabilitation applications are extensive. According to [32], despite some differences in range of motion (ROM) of body articulations, reproducibility of Kinect recordings is comparable to marker-based systems. This feature is especially interesting when considering biomechanical evaluations, since pre-post assessments must be reliable, repeatable, and comparable. In [33], it was found that Kinect-based 3D reachable workspace analysis of the upper limb provides sufficiently accurate and reliable results as compared to motion capture systems; the same was found in [34], evaluating shoulder ROM. On the contrary, in [35], consistent discrepancies were found in evaluating shoulder angles, even if such results are in general contracting the majority of the works.

In 2014, the Kinect V2 sensor (second generation of the Kinect) was released, featuring a more precise 25-joint tracking. As its predecessor, Kinect V2 was applied to the rehabilitation field. In our review of the literature, a consistently lower number of studies was found using Kinect V2 in respect to Kinect. Some Kinect V2-based applications rely on the interaction with simple virtual environments or games that hide functional rehabilitation behind gaming approaches [36]. Kinect V2 was chosen to study postural control [37], concluding that the results were generally comparable to the ones obtained with marker-based systems. Applied to gait analysis, other studies suggested that the Kinect V2 has the capability to measure effectively selected spatiotemporal gait parameters for healthy adults [38]. According to [39], the Kinect V2 sensor is able to offer state-of-the-art head pose estimation accuracy in real time and without the need for calibration. Despite the increased accuracy and the tracking algorithms, a limited number of studies reported the use of the Kinect V2 sensor as an instrument for providing evaluations of the biomechanics of the movement and the quality of motor control of the upper limb. In [40], the articular range of motion was evaluated, concluding that Kinect V2 precision is acceptable for clinical applications or evaluation of motor performances of the upper limb. A study [41] proposed preliminary results on Kinect V2 as a tool for evaluating the biomechanics of the upper limb, indicating, with preliminary results, that Kinect V2 might be suitable for patient evaluation by providing coherent assessment in respect to clinical scales. However, as previously mentioned, a reduced number of studies evaluating Kinect V2 performances on patients in comparison to golden standard marker-based system methods were found. In [42], authors found “excellent agreement between Kinect and Vicon gold standard as well as retest reliability for a variety of kinematic parameters extracted from different motor tasks of clinical interest” on a quite wide sample of neurological patients. In [43], a detailed analysis is performed to verify the suitability of Kinect V2 as a tool to evaluate rehabilitation of the upper limb, coming to the encouraging conclusion that “the device is suitable for the rehabilitation application.” It should be underlined, however, that several studies indicate adequacy of the sensor on healthy people tracking aimed at motor evaluations, suggesting its application to pathological movement: comparing Kinect V2 and a marker-based system, in [42], it was found that “in summary, most clinical parameters showed high absolute agreement and no systematic bias between systems. The parameters that showed moderate absolute agreement mostly showed high consistency agreement as well”. Similar results were found in Parkinson disease assessment [44], in gait analysis and evaluation [45], and for dynamic movements in rehabilitation scenarios [46]. In [47], a small cohort of neurological patients is clinically evaluated by the means of Kinect V2. A recent study [48] assessed Kinect V2 as a tool for evaluating spinal muscular atrophy patients, matched with healthy controls, concluding that “Microsoft Kinect V2 sensor has the potential of being developed into a complementary output measure as it provides reproducible, objective and detailed information of body point motion.” In addition, [48] addresses the issue of Kinect V2 repeatability, with promising results to be furtherly investigated. In [49], Kinect V2 was used to record the kinematics of the upper limb as trigger for functional electrical stimulation of a robotic setup aimed at providing assistance in the home environment, confirming high confidence on system reliability. Furthermore, Microsoft datasheets claim that Kinect V2 features “improved body tracking (the tracked positions are more anatomically correct and stable and the range of tracking is broader), improved depth sensing (with higher depth fidelity and a significantly improved noise floor, the sensor gives you improved 3D visualization, improved ability to see smaller objects and all objects more clearly, and improves the stability of body tracking), 1080p color camera (30 Hz), New active infrared (IR) capabilities 512 × 424 30 Hz.” Considering that (1) all the technical specifications are superior to the ones of Kinect and that (2) Kinect is considered as valuable for motor evaluation, it is licit to assume that Kinect V2 is a valuable system too. Furthermore, a detailed study, not oriented to rehabilitation, underlined that Kinect V2 performances are evidently higher than the Kinect Ones [50]. Embedded algorithms for joint tracking make it one of the most valuable, despite affordable, substitute of marker-based systems.

**Figure 1:**
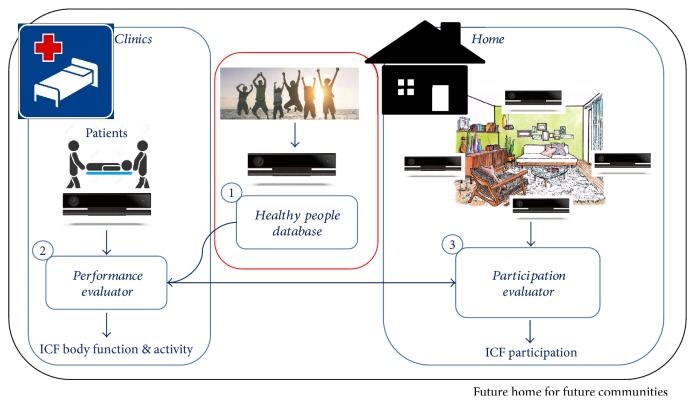
The framework of the Future Home for Future Communities (FHfFC) research project. Label 1 indicates the module called “Healthy People Database (HPD)” that creates reference normative data for the “performance evaluator module (PEM)” (labeled 2). Thanks to the reference provided by HPD, PEM can determine evaluation of ICF body function and activity domains. Label 3 indicates the participation evaluator (PE) that uses PEM in an integrated module that describes the interaction of the patient to real life actions in the home environment. In this study, the red-circled part of the framework is assessed: Healthy People database.

In the framework of the research project “Future Home for Future Communities (FHfFC),” funded by the Italian National Research Council and the Italian Lombardy Region (framework agreement), in the light of the literature and of the previous comments, Kinect V2 is used as an evaluation and monitoring tool on neurological patients both in the clinical and home environments as synthetized by the scheme in Figure 1. Kinect V2 is considered as an affordable tool capable of quantified assessment of ICF body function and activity capabilities in the clinics, while, at home, it will be used also for the assessment of the participation domain.

A simple but consistent upper limb functionality evaluation module, coupled with low-cost technologies, could provide valuable improvements for both clinical and home therapies and monitoring. In the clinics or in little rehabilitation centers and laboratories, it could support or substitute clinical scale evaluation, providing low-cost and time-saving motor assessments. At home, during the execution of unsupervised domestic training or during daily-life activities, the evaluation module could be helpful for setting training difficulty when integrated in virtual applications, give feedback to the patients for motivation, and monitor the quality of the training and life at a distance. This study represents the first stage of FHfFC. Referring to Figure 1, the focus was on body function and activity domains, by addressing the functionality of the upper limb and in particular of the proximal joints (the shoulder and elbow).

**Figure 2:**
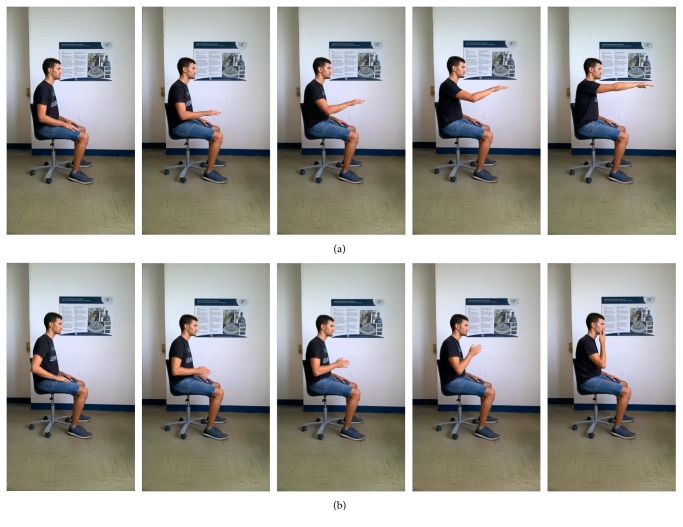
(a) The reaching movement (RM) and (b) hand-to-mouth movement (HtMM).

A previous work [51] presented a marker-based biomechanical evaluation protocol to be used in the clinical environment, based on two functional gestures: the reaching movement (RM, depicted in Figure 2(a)) and the hand-to-mouth movement (HtMM, depicted in Figure 2(b)). The RM movement was chosen as it is fundamental for autonomy in daily-life activities, because it simultaneously (1) involves multijoint coordination, (2) involves capability of elevating the arm against gravity, (3) allows to reach for desired objects, and (4) allows interaction with the environment. The HtMM movement was considered since it involves the capability of bringing objects towards the body, representing a motor primitive for eating, dressing, and many other activities of daily life and a natural complementation to the RM movement. The coordinated capability of performing RM and HtMM allows a wide exploration of the workspace of the upper limb and a purposeful interaction with the environment. Such choice of motor tasks also stresses the capability of moving against gravity, which is one of the focusing capabilities that might strongly differentiate the possibility of interacting with the environment for poststroke patients.

Supported by promising evidences found in the literature, especially when considering healthy people, in this paper, Kinect V2 is presented as a tool for the evaluation of the motor performances of neurological patients during the execution of RM and HtMM. In order to achieve such result, a detailed knowledge of the performances of healthy subjects, as measured with the Kinect V2 sensor, is requested. Thus, this paper investigates and characterizes the presence of differences in performances of healthy subjects during the execution of RM and HtMM. In fact, the analysis was performed with the aim of properly organizing healthy subjects' data as a reference for evaluating motor performances of neurological patients. In order to obtain such results, the acquisition of more knowledge on the motor performances on healthy subjects in daily-life gestures, measured with the Kinect V2, is needed. When a patient will be recruited for assessment, his/her motor performances would be crossed with the reference data that refer to the proper subset of healthy subset (the group to which the patient would belong if he/she would not be impaired). The analysis was conducted considering three main macrosubsets of healthy subject clustering that were considered as possible sources for differentiation in healthy subjects. The first two considered subsets were the dominant and nondominant limbs. The investigation was also conducted dividing the population by gender and by three age subsets (young adults, middle-aged people, and senior), by testing differences for the dominant and nondominant limbs. Depending on gender, age, and impaired limbs, subsets of comparative healthy subjects' performances can be arranged. Whereas statistical differences could not be spotted, different subsets could be integrated to enlarge the comparative datasets.

## 2. Materials and Methods

### 2.1. Objectives

The aim of the study was to investigate the performances of healthy subjects in RM and HtMM movements with the Kinect V2 commercial, low-cost sensor. *The main investigation was conducted by clustering a cohort of healthy subjects depending on their limb dominancy*, *gender and age*, *and testing whether motor performances are influenced or not by such characteristics.* Following the analysis, a normative database of healthy subjects' performance will be build, in order to provide reference ranges of biomechanical performances to be compared to neurological subjects' performances.

### 2.2. Setting

The study took place at the Institute of Industrial Technologies and Automation (ITIA) of the Consiglio Nazionale delle Ricerche (CNR), Milano, Italy. Recruitment of participants took place at the Institute of Industrial Technologies and Automation (ITIA) of the Consiglio Nazionale delle Ricerche (CNR), Milano, Italy.

### 2.3. Participants

Criteria for eligibility were being neurologically and orthopedically intact. A cohort composed of 78 healthy subjects (47 males, 31 females, mean age 41.77 ± 19.29), unaware of the purpose of the study, was enrolled for the experiment, after giving informed consent. Subjects' characteristics are summarized in Table 1. The experiment was conducted according to the Declaration of Helsinki. Subjects were subdivided into groups depending on their limb dominancy, gender, and age.

### 2.4. Experimental Setup

**Figure 3:**
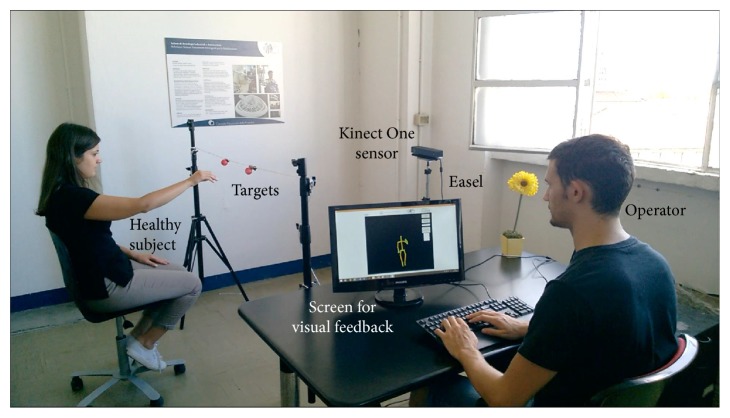
The experimental setup.

During the trials, the experimental setup (portrayed in Figure 3) was composed of the following:

- A Microsoft Kinect V2 sensor version 2.0, mounted on an easel and placed at about a 2.0 m distance from the torso of the subject
- In-house software for online feedback and data logging
- A PC with Microsoft Windows 8.1, USB 3.0, and Microsoft Kinect One S.D.K. version 2.0
- A screen providing online visual feedback to the operator to visually monitor the correctness of the kinematic tracking

### 2.5. Motor Gestures

The enrolled subjects performed the reaching against gravity movement (RM) and hand-to-mouth movement (HtMM) according to the protocol described in [51]. Subjects stood comfortably on a chair, with their back straight. They were asked not to move their torso during the experiment and to perform the motor tasks only by limb motion. A target for the RM was set at shoulder height, indicating the point toward which subjects had to point to. The target of the HtMM was the mouth itself. Subjects were requested to perform the repetitions of the gestures at natural, comfortable, self-selected speed, with no pauses between one repetition and the following one. The starting position was with the elbow flexed of about 90°, with pronated hand leaning on the thigh. Each subject performed four acquisitions: RM and HtMM gestures, both performed with their dominant and nondominant limbs. Twelve repetitions of the motor task were performed per acquisition.

Microsoft Kinect V2 sensor was used to record movement execution. In total, 78 subjects executed 4 acquisitions for a total of 312 records.

Microsoft Kinect V2 includes embedded libraries for skeleton tracking. They allow the tracking of human movement but do not allow defining other reference points other than the articular joint centers. Consequently, without the inclusion of specific image segmentation algorithms, a fine characterization of the interaction with an object is not possible. However, as stated in [52], motor control analysis and knowledge acquired in tests on “standardized” reaching movements (without physical interaction with a grasped object) can be considered as representative of “natural” reaching movements, involving physical interaction with real objects. Consistently, the use of the Microsoft Kinect V2 sensor for the analysis of reaching movements is a reasonable (and cost-effective) choice.

Microsoft Kinect V2 is designed mainly to record people facing the sensor while standing. The Kinect V1 also provided a seated tracking mode that was conceived to simplify and optimize seated positions, which is not implemented in Kinect V2. A study revealed some limitations in detecting specific postures and body angles [53], but such angles are not investigated in this study. Authors did not notice remarkable difference between standing and seating while executing upper limb tasks, and the tracking was always adequate, considering that only upper limb data were considered. Some studies reported similar conclusions about adequateness of the Kinect V2 used in seated or comparable positions, while examining tasks including squatting [46], sitting [42, 47], or even while seated in interaction with robotic devices [49]. Considering future applications on neurological patients (who may not be able of standing autonomously), a requirement for the study was to acquire normative data while in seated position.

### 2.6. Data Sources and Measurements

Data were recorded and logged with an in-house C#-developed software for visual feedback and data acquisition. Offline analysis was performed by the means of an in-house-developed Matlab software.

3D joint tracking data of shoulder, elbow, wrist, and time labels were recorded and logged for offline analysis. In order to eliminate noise, tracking data were low-pass Butterworth filtered, 3rd order, cutoff frequency 6 Hz. An algorithm for automatic phase detection was implemented. It was needed to separate the forward phase of the movements by the backwards one. All the outcome measures were performed in the forward phase.

### 2.7. Outcome Measures

The recorded data were used to compute the following outcome measures (according to a protocol presented and described in detail in previous studies [28, 29, 41]):

1. Kinematics and Range of Motion
  - Execution time (*T*) (s)
  - Shoulder elevation angle (SE) (*°*)
  - Elbow flexion and extension angle (EF) (*°*)
  The above-listed parameters account for movement kinematics (timing and range of motion) at the end of the forward phase of the movement.
2. Dynamics
  - Shoulder elevation torque (ST) (Nm)
  - Shoulder effort index (SEI) (Nms)
  The above-listed parameters account for movement dynamics (torques at the end of the movement and cumulative torque during the forward phase). They were obtained by the means of a simple biomechanical model of the upper limb accounting gravitational and inertial contributions to articular torques. The biomechanical model associated to each body segment a mass obtained from anthropometric tables starting from the body mass. Segment masses were located in the segment barycenter identified by anthropometric tables.
3. Motor Control and Motion Quality
  - Normalized jerk (NJ) (a dimensional number) [28]
  - Coefficient of periodicity (ACP) (a dimensional number) [28]
  The above-listed parameters account for movement quality of execution, representing, respectively, the smoothness (normalized jerk) and the repeatability of the acceleration profile (coefficient of periodicity) of the forward phase.

The protocol was designed to be a simplified, synthetic version, made of a selection of parameters computed in clinical environments during robotic therapies [54].

Shoulder evaluations mainly characterize the RM, while elbow evaluations are related mainly to the HtMM. This is relevant especially for healthy subjects that execute “correctly” the motor tasks. However, patients might show impaired motor functions and compensatory motor strategies (e.g, to bring the hand to the mouth, they abnormally elevate the shoulder). As reference values for the assessment of abnormal motor strategies, both shoulder and elbow evaluations were computed for RM and HtMM.

The present study aims at analyzing the influence of limb dominancy, gender, and age on the biomechanics of daily-life gestures, when measured with a Kinect V2 sensor.

### 2.8. Study Design

In the *main investigation*, subjects were clustered into three groups of data subsets, depending on the following criteria: (1) limb dominancy: the dominant/nondominant limb; (2) gender: males/females; (3) age: young adults/middle aged/senior. For each of the three macrogroups (dominancy, gender, and age), each subset was tested in comparison to others on each variable of the biomechanical assessment. Means and standard deviations were computed along with *p* values of the comparisons. The total are as follows:

- Dominancy test: 14 tests (2 gestures × 7 variables of the biomechanical assessment)
- Gender test: 28 tests (2 gestures × 2 limbs × 7 variables of the biomechanical assessment)
- Age test: 28 tests (2 gestures × 2 limbs × 7 variables of the biomechanical assessment)

Two aspects were expected to have particular relevance on results: the effect of age and the quality of tracking on derivative quantities (velocity, acceleration, and jerk).

Since motor control indexes are based on derivatives of the position, they are more sensible to measurement errors (see Discussion for details). For this reason, a more detailed analysis was conducted on NJ and ACP to test the dependency of such indexes in respect to movement time, depending on age. The same analysis was performed in a previous study [51] on healthy people using a marker-based system. It is worth to underline that in [51], no procedures for the identification of the joint center were performed and markers were used as representative of the shoulder, elbow, and wrist. Kinect V2 provides instead an estimation of the joint center starting from RGB and depth stream data. Consequently, results in [51] and in the present study are not directly comparable. Subjects were divided into four subsets (young adults, middle aged, senior, and all). Regression curves (*T*, NJ) and (*T*, ACP) were analyzed for both RM and HtMM, NJ and ACP, and for each age subset and considering all the subjects together, for a total of 32 regression curves (2 gestures × 2 limbs × 2 motor indexes × 4 subsets each). Regression curves were compared to the trends found in the abovementioned study.

### 2.9. Statistics

In the main investigation, for each dependent variable belonging to the evaluation protocol and to a specific data subset to be tested, the normality of the distribution was assessed using the Kolmogorov-Smirnov normality test.

Normality and statistical tests were performed with the Python scripting language, using the scipy.stats (science python, Matlab like) package.

In case that some data subsets deviated from normality, as it had happened indeed in some cases, authors decided to use nonparametric tests for comparisons, also considering the different wideness of samples of several data subsets.

For testing the biomechanical differences related to limb dominancy (population sample: dominant/non dominant limb—78 samples each) and for testing the biomechanical differences related to gender (population sample: males/females—47 M/31 F), along the single variables that are part of the assessment, the nonparametric Mann–Whitney *U* test was used.

For testing the biomechanical differences related to age (population sample: young, 36; middle, 19; and old, 23), along the single variables that are part of the assessment, the Kruskal-Wallis nonparametric test was used.

The alpha-error significance level was set to 0.05.

For the further investigation on NJ and ACP, to test the (*T*, NJ) and (*T*, ACP) dependencies, regression curves were found and Pearson's correlation coefficients were computed.

## 3. Results

### 3.1. Participants

All participants concluded the test.

### 3.2. Limb Dominancy

The results of the Mann–Whitney *U* test, comparing the dominant and nondominant limb motor performances in RM and HtMM, are reported in Table 2. No statistical difference was found in any of the parameters of the evaluation (*p* > 0.05) except for shoulder elevation (*p* = 0.01) in the RM. In case *p* < 0.05, indicating a statistically significant difference between the groups, data subsets were highlighted in bold.

### 3.3. Gender

The results of the Mann–Whitney *U* test, comparing motor performances of male and female upper limbs, in RM and HtMM, are reported in Table 3 (RM) and Table 4 (HtMM). No statistical difference was found in any of the parameters of the evaluation (*p* > 0.05) in the RM. Significant differences were spotted in HtMM, nondominant limb, in shoulder elevation (*p* = 0.038), elbow flexion (*p* = 0.004), shoulder torque (*p* = 0.022), shoulder effort index (*p* = 0.007), and acceleration coefficient of periodicity (*p* = 0.029).

### 3.4. Age

The results of the Kruskal-Wallis test, comparing young, middle, and elder limb motor performances, in RM and HtMM, are reported in Table 5 (the dominant limb) and Table 6 (the nondominant limb). Elderly people show slower movement time (*p* = 0.014) in RM, dominant side. In HtMM, dominant side, elderly people show slower movement time (*p* = 0.030), higher shoulder effort (*p* = 0.045), and higher normalized jerk (*p* = 0.024). No significant differences related to age were found on other parameters (*p* > 0.05).

### 3.5. Age Investigation: Motor Control Regressions

**Figure 4:**
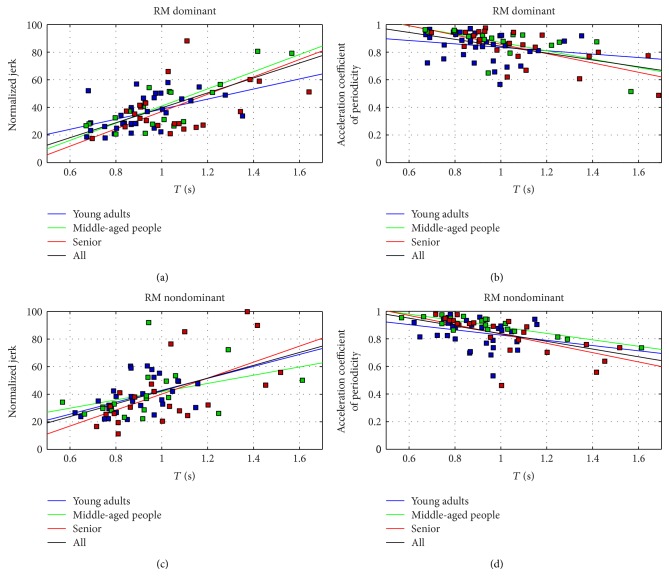
RM: regression curves for normalized jerk and acceleration coefficient of periodicity for age groups.

**Figure 5:**
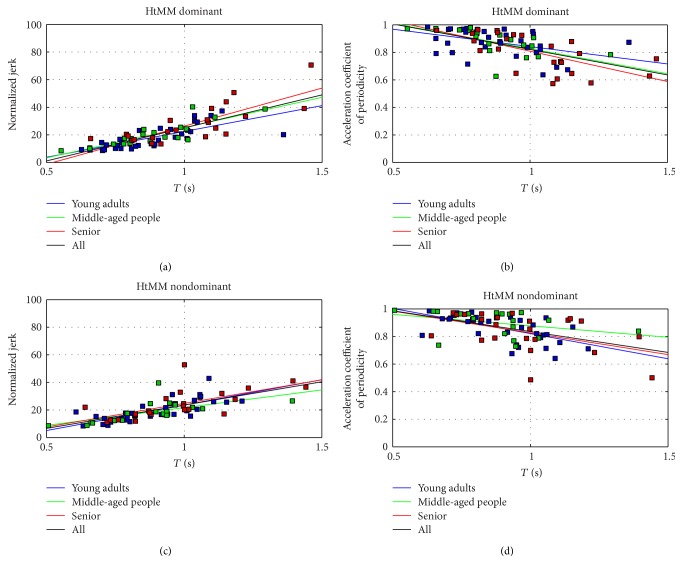
HtMM: regression curves for normalized jerk and acceleration coefficient of periodicity for age groups.

Tables 7 and 8 summarize the result of the statistical tests in RM and HtMM, respectively, for NJ and ACP. Regression coefficients (*R*) and *p* values are reported. Figures 4 and 5 report regression curves and data scatter.

## 4. Discussion

### 4.1. How Limb Dominancy Affects Performance in RM and HtMM?

Significant differences in motor performances could be expected when evaluating the performances of the dominant versus nondominant limbs, since it is well known that the dominant and nondominant limbs have a tendency to specialize in dynamical and static motor tasks, respectively [55]. Furthermore, the dominant arm can achieve more varied and flexible control over movement trajectories, while accuracy and precision are comparable [56]. However, such differences would be particularly expected in relation to highly demanding motor tasks (hard to complete or very fast, engaging, and not already known) or in fine control (related, e.g., to hand or finger dexterity [57]), “in favor” of the dominant limb, rather than in daily-life, well-known gestures as RM and HtMM.

Consistently with such premises, a previous study, performed with the marker-based system on RM and HtMM, found no difference in performance between the dominant and nondominant limbs [51].

Accordingly, in this study, significant difference between the dominant and nondominant limbs was found nor in RM nor in HtMM (for all parameters, *p* > 0.05), underlying high similarity in motor performances for dominant and nondominant limbs in our sample of healthy subjects. The only exceptions are represented by elbow torque (*p* = 0.025) and the small (~3°) but significant difference in shoulder elevation (SE) in the RM (dominant = 103.71° ± 8.06°; nondominant = 106.32° ± 7.64°, *p* = 0.01). Since SE characterizes the RM, concurring to shoulder flexion during the motor gesture, such difference has to be discussed properly. A possible explanation might be related to the fact that the dominant limb elevates slightly less, indicating more precision in executing the RM, without exceeding in the range of motion. The authors checked the orientation of Kinect V2 before every acquisition and consequently introduced no systematic errors. Furthermore, intratrial shoulder elevation standard deviation, related to the same performer, may have higher intrasession deviation if compared to the difference between dominant and nondominant RM performances. This issue suggests that SE difference might lie in physiological motor control variability, slightly in favor of the precision of the dominant limb, which is an acceptable, despite unexpected, result. Lastly, all the other performance indexes did not significantly differ, strongly suggesting the equivalence between the dominant and nondominant sides. Authors conclude that, in general, no remarkable difference was spotted between dominant and nondominant execution of RM and HtMM, probably due to the fact that the examined motor gestures did not stress healthy subjects' capabilities. Probably, more demanding gestures would underline the dynamic specialization of the dominant limb [55], promoting the emergence of remarkable performance asymmetries.

### 4.2. How Gender Affects Performance in RM and HtMM?

As for dominancy tests, significant differences in motor performances between males and females were not expected since RM and HtMM are daily-life, well-known gestures and not highly demanding motor tasks, not requiring specific capabilities (e.g., in terms of strength that might privilege males over females). In this study, no significant differences were found in the RM (for all parameters, *p* > 0.05), underlying the high similarity in motor performances for male and female limbs in our sample of healthy subjects. In the HtMM, nondominant side, statistically significant differences were instead found in all the assessments related to the shoulder: shoulder elevation (*p* = 0.038), shoulder torque (*p* = 0.022), and shoulder effort index (*p* = 0.007) are all lower in females.

As remarked before, significant differences in shoulder elevation, torque, and effort were spotted between males and females. In particular, our sample of data underlines a tendency of males to elevate the shoulder slightly more, with repercussions on higher exerted torques (higher gravitational and inertial loads are requested to the shoulder) and shoulder effort. Such tendency is also reflected by a slight decrease of the acceleration coefficient of periodicity (*p* = 0.029), probably because more variability in the motion law is introduced due to the increased range of motion that makes the HtMM closer to a multijoint task in males rather than in females. This hypothesis is supported by the fact that, in females, elbow flexion at the end of the movement is significantly higher (*p* = 0.004), indicating that the target is reached with a quasi-monojoint movement. Such results suggest that, in the HtMM, males and females show remarkable motor differentiation. Interestingly, the same tendency is confirmed on the dominant side, where all the trends found on the nondominant side are not significant, but still observable. Probably, in the dominant side, motor control is more effective and motor control defects are reduced. Authors' finding is supported by recent studies that suggest that females have better position sense and precision associated to shoulder movement [58]. This difference might be explained also by the fact that a wider amplitude of the torso and stronger musculature, typical of males, might force, at the beginning of the movement, a slight shoulder intrarotation that, as a repercussion, leads to a gesture composed of more remarked shoulder elevation and abduction in males.

### 4.3. How Age Affects Performance in RM and HtMM?

Age has observable outcomes on the biomechanical performances of the two proposed gestures. In particular, elderly people show slower execution times (*p* = 0.014) in RM, dominant side, and HtMM, dominant side (*p* = 0.030), in respect to the young and middle groups. A similar tendency appears in both gestures, even in the nondominant side. Despite that a fast, physiological execution time is not strictly requested for the successful performing of a motor task, execution times are a fundamental aspect of motor control, even if the principles underlying its formation remain little known. Previous studies investigated the role of time in motor control to uncover what optimality criterion underlies human motor behavior. In the literature, a “cost of time” theory was introduced [59], which asserts that the time elapsed until action completion entails a cost, thereby making slow moves nonoptimal. Furthermore, for a proper exploitation of the dynamical properties of the limb [41], “quite fast” movement execution can be considered as optimal, since it reduces peak torques. In the literature, a pronounced increase in movement duration with age is seen on a variety of tasks. Movement slows with age by as much as 15–30%. This slowing appears in part to be strategic because older adults emphasize movement accuracy at the cost of movement speed. Slower information processing may also affect motor performance in a nonspecific, global fashion due to an increase in neural noise and other synaptic changes.

The results are corroborated by other assessments related to gesture dynamics: shoulder effort is higher in elderly people in HtMM, dominant side (*p* = 0.045), and motor control (normalized jerk in HtMM, dominant side, is higher indicating less smoothness, *p* = 0.024). The same tendency is confirmed, while not being significant, in the smoothness of the HtMM in the dominant side, while no difference among groups is spotted in RM. Being related to motor performance, also smoothness differences could be expected when evaluating the performances of the dominant versus nondominant limbs. Such difference was not detected, probably because the examined motor gestures did not stress healthy subjects' capabilities. However, it is worth to mention that RM and HtMM may actually be very demanding for neurological patients. High repeatability is a feature typical of skilled motor control.

Such results can be explained by the fact that elderly people are less selective in the activation of cortical areas and, consequently, their performance tend to decrease, even in simple daily-life gestures [60]. At the same time, when executing motor tasks, elderly people show activations of supplementary areas that are not strictly related to the movement to be produced, indicating less focused motor control and cortical area activations [60]. In general, older adults exhibit involvement of more widespread brain regions for motor control than young adults, particularly the prefrontal cortex and basal ganglia networks. Unfortunately, these same regions are the most vulnerable to age-related effects. Older adults show deficits in coordination of bimanual and multijoint movements. For example, movements become slower and less smooth when older adults move their shoulder and elbow joints simultaneously as opposed to performing single joint actions [60].

The role of smoothness in motion control was widely investigated in the literature. Smooth movement execution is coupled to quality of motor control and is typical of physiological motor patterns. High smoothness is also typical of skilled motor performers (such as sport practitioners) and of fast-executed and trained movements. Consequently, it is not surprising that Caimmi [61] and Kimura [62] reported that young subjects tend to achieve higher smoothness in respect to elderly people in daily-life gestures. Low smoothness, instead, characterizes motor patterns of neurological patients [63]. Patients' motor recovery was suggested to be related to the smoothness of the training movements executed during the therapy [64], clearly indicating the crucial role played by smoothness in describing motor performances. Smoothness is usually measured with normalized jerk or number of velocity peaks within a repetition of the motor task.

Previous studies investigated the repeatability of the motion law in daily-life gestures on healthy subjects [41] and patients [61], not only concluding that such index is sensible both to being healthy and a patient but also indicating differences even among patients. The investigation of such issue, on a limited sample of healthy subjects and patients, leads to the conclusion that Kinect and Kinect V2 sensors, despite lower sampling frequency, could cluster differently healthy subjects and patients and even distinguish between different levels of motor impairment. The present study is meant also to investigate such issue on a wider sample of people and under challenging conditions for the sensibility of the Kinect V2 sensor. In fact, in well-known daily-life gestures, performed by healthy subjects, motor differences are expected to be of minor relevance, being more challenging to be detected.

As shown in previous studies [51], normalized jerk and acceleration coefficient of periodicity represent interesting parameters to be assessed to detect motor capability. Specific trends in NJ and ACP were found on healthy subjects depending on age, according to the clustering proposed in the present study. In RM and HtMM, for both the dominant and nondominant limbs, a regression involving execution time and NJ/ACP was performed (for a total number of 32 regressions: 4 groups—young adults, middle aged people, senior, and all) × 2 movements (RM and HtMM) × 2 limbs (dominant and nondominant) × 2 performance indexes (NJ and ACP). In [51], significant moderate to high positive correlation for (*T*, NJ) and negative correlation for (*T*, ACP) were found for all the regressions (*p* < 0.05, except one). Furthermore, it was observed that each linear regression was associated to a different slope. Young and middle-aged subjects had in general lower slopes, suggesting that, given an execution time, young and middle-aged subjects could achieve higher performance indexes in respect to older ones.

The same regressions were performed in this study with the Kinect V2. Very interestingly, 16 over 16 regressions were positive for (*T*, NJ) and 16 over 16 regressions were negative for (*T*, ACP), as found with marker-based systems. In particular, 8 over 8 correlations involving all subjects (all) showed strong statistical significance (*p* < 0.001), associated to a *R* correlation that ranged between 0.49 to 0.81 (from moderate to high). Such result indicates that the Kinect V2 correctly and quite solidly captures basic trends associated to NJ and ACP.

Furthermore, 28 over 32 correlations were statistically significant (*p* < 0.05) with the exception of the middle-aged group (NJ, RM nondominant, *p* = 0.088; ACP, HtMM nondominant, *p* = 0.135) and the young adults (ACP, RM dominant, *p* = 0.268; ACP, RM nondominant, *p* = 0.154). All the significant correlated regressions were associated to a moderate or high value of *R* (0.50 < *R* < 0.89).

The slope associated to the regression lines can be considered as a measure of the motor control capability of subgroups of subjects. Higher slope is associated to major degradation in performance (lower repeatability, lower smoothness). In RM and HtMM, Kinect V2 always individuates higher slopes associated to seniors' motor performance, in respect to young and middle-aged subjects, both in NJ and in ACP. The ACP in HtMM nondominant limb represents the only exception; in this case, motor performances of young adults are comparable (slightly higher) to those of the senior group. It should be remarked that a rigorous interpretation of the regression curves might lead to believe that seniors have better control parameters in respect to young and middle-aged people for fast movements, and vice versa for slow movements. Such interpretation is actually misleading, since the slope should be observed only in the range of values that are connected to that specific group performances; in fact, seniors have in general slower execution times.

Globally, results are encouraging. While marker-based devices should still represent the more reliable and precise instruments for investigating motor control, these results are of primary importance, since they indicate that, despite limitations in the sampling frequency, Kinect V2 can detect primary features of motor control as more expensive motion capture systems do (even if with the discussed limitations).

### 4.4. Limitations

A worth-mentioning limitation of the Kinect V2 sensor is the sampling rate that is set to 30 Hz. Marker-based systems, instead, provide higher sampling frequencies (100 + Hz). While such limitation does not affect the evaluation of the range of motion considering the quite slow dynamics of the proposed gestures, it may affect the computation of motor control indexes that rely on velocity, acceleration, and jerk. Furthermore, Kinect V2 provides an estimation of joint centers obtained by segmentation of RGB and depth stream. As discussed in the introduction, many comparisons with marker-based systems are available in the literature describing that Kinect and Kinect V2 are in general adequate tools for human motion analysis. However, such characteristics should be considered to critically interpret the results.

As a general remark, the sensibility of the proposed methodology (based on RM and HtMM) proved not to be particularly high on healthy subjects in distinguishing motor performances, while it is for neurological patients since it involves demanding gestures for impaired people. Such result was expected since daily-life, well known, and quite rapid gestures are performed mainly in feedforward control [65]. Thus, such gestures “belong” to the hard-coded, well-known set of mastered control abilities, as suggested by the Schema Theory of Motor Learning [16]. However, it was anyway possible to detected significant differences on motor control parameters. Significant differences were found especially when considering young and middle-aged groups in comparison to the old group, and even male and female motor performances differ in some aspects. Such findings will allow a proper mapping of the subsets of healthy people data into reference data for neurological patients.

Moreover, results indicate that Kinect V2 is capable of reproducing trends observed with marker-based systems and can be used as a valuable and affordable tool for healthy people and neurological patient evaluations. Furthermore, the methodology was designed considering the needs of neurological patients that, depending on the severity of their motor impairment, may consider RM and HtMM as highly complex and challenging to perform. For neurological patients, the RM and HtMM tasks include multijoint, coordinated movements against gravity, making them challenging enough to trigger the execution of feedback control, thus activating the process of motor learning and neuroplasticity typical of motor relearning [65].

## 5. Conclusion

A Kinect V2, coaching-based approach for the evaluation of motor performances of neurological patients was proposed. A healthy subjects' database was built and used as reference for statistical analysis of neurological patients' performances, revealing significant differences in motor execution between the young and middle subgroups in respect to elderly people and between males and females. Such findings will be the basis reference for affordable, home-oriented, easy-to-perform motor evaluation on neurological patients.

Further studies will use the proposed methodology for the evaluation of neurological patients and in particular for low-cost, easy-to-perform, and easily administrable motor assessments, even outside the clinical environment, as proposed by the Riprendo@Home and the Future Home for Future Communities research projects. The correlation of the proposed methodology with the upper limb parts of the most used clinical scales (such as the Fugl-Meyer assessment) will be investigated, to evaluate how the Kinect V2-based assessment could be used as an integration or sometimes as a substitute, of the clinical scales. Lastly, the evaluation module will be an important tool to be integrated in home-oriented solutions for rehabilitation, as a simple tool for motor evaluation or as biomechanical support to tune the training process both at home and clinical environments in the framework of the Future Home for Future Communities research project.

## Figures and Tables

**Table 1:** Participants.

Participants								
Males	Females	Right is dominant	Left is dominant	Age < 35	34 < age < 51	Age > 50	Mean age	Total
47	31	72	6	36	19	23	41.77 ± 19.29	78

**Table 2:** Dominancy subsets.

Dominancy	Reaching movement			Hand-to-mouth movement		
Parameter	Dominant	Nondominant	*p* value	Dominant	Nondominant	*p* value
*T* (s)	0.99 ± 0.22	0.96 ± 0.24	0.094	0.93 ± 0.18	0.92 ± 0.18	0.387
SE (°)	**103.71** ± **8.06**	**106.32** ± **7.64**	**0.01**	35.87 ± 13.33	38.37 ± 14.26	0.19
EF (°)	15.07 ± 7.41	16.74 ± 7.14	0.052	139.51 ± 9.72	137.63 ± 9.39	0.227
ST (Nm)	7.05 ± 0.55	7.12 ± 0.58	0.28	4.24 ± 0.98	4.43 ± 1.12	0.114
SEI (Nms)	5.53 ± 1.51	5.5 ± 1.67	0.363	3.33 ± 1.02	3.53 ± 1.22	0.218
NJ	41.27 ± 25.18	42.09 ± 21.19	0.252	21.81 ± 11.16	22.86 ± 20.39	0.473
ACP	0.83	0.84	0.386	0.83	0.85	0.262

**Table 3:** Gender subsets: reaching.

Gender	Reaching dominant limb			Reaching nondominant limb		
Parameter	Males	Females	*p* value	Males	Females	*p* value
*T* (s)	0.99 ± 0.22	0.99 ± 0.22	0.479	0.96 ± 0.21	0.95 ± 0.27	0.277
SE (°)	103.41 ± 7.19	104.16 ± 9.19	0.279	106.04 ± 6.13	106.73 ± 9.43	0.448
EF (°)	15.3 ± 7.6	14.73 ± 7.09	0.416	17.56 ± 7.87	15.52 ± 5.67	0.202
ST (Nm)	7.02 ± 0.55	7.08 ± 0.55	0.265	7.19 ± 0.62	7.02 ± 0.5	0.109
SEI (Nms)	5.51 ± 1.5	5.56 ± 1.52	0.436	5.59 ± 1.48	5.35 ± 1.89	0.149
NJ	42.05 ± 29.44	40.1 ± 16.92	0.307	42.51 ± 22.95	41.48 ± 18.25	0.326
ACP	0.82	0.86	0.085	0.82	0.86	0.235

**Table 4:** Gender subset: hand-to-mouth.

Gender	Hand-to-mouth dominant limb			Hand-to-mouth nondominant limb		
Parameter	Males	Females	*p* value	Males	Females	*p* value
*T* (s)	0.93 ± 0.14	0.92 ± 0.23	0.206	0.93 ± 0.16	0.89 ± 0.21	0.064
SE (°)	36.06 ± 13.11	35.58 ± 13.63	0.388	**40.92** ± **13.57**	**34.59** ± **14.43**	**0.038**
EF (°)	138.4 ± 9.99	141.15 ± 9.06	0.08	**135.24** ± **9.16**	**141.16** ± **8.58**	**0.004**
ST (Nm)	4.33 ± 1.0	4.09 ± 0.94	0.176	**4.67** ± **1.05**	**4.09** ± **1.15**	**0.022**
SEI (Nms)	3.46 ± 0.97	3.14 ± 1.07	0.117	**3.78** ± **1.07**	**3.15** ± **1.32**	**0.007**
NJ	22.44 ± 10.0	20.88 ± 12.64	0.123	24.73 ± 25.34	20.08 ± 8.16	0.223
ACP	0.83	0.84	0.232	**0.83**	**0.88**	**0.029**

**Table 5:** Age subset: reaching.

Age	Reaching dominant limb				Reaching nondominant limb			
Parameter	Young	Middle	Senior	*p* value	Young	Middle	Senior	*p* value
*T* (s)	**0.91** ± **0.15**	**1.01** ± **0.22**	**1.09** ± **0.25**	**0.014**	0.89 ± 0.13	0.95 ± 0.24	1.07 ± 0.31	0.074
SE (°)	103.28 ± 5.96	101.54 ± 5.38	106.17 ± 11.37	0.14	105.73 ± 5.78	105.3 ± 4.37	108.06 ± 11.15	0.47
EF (°)	14.7 ± 7.97	13.9 ± 5.63	16.59 ± 7.57	0.602	15.72 ± 6.91	17.22 ± 6.78	17.89 ± 7.53	0.475
ST (Nm)	7.0 ± 0.6	7.04 ± 0.54	7.12 ± 0.48	0.742	7.1 ± 0.56	7.27 ± 0.51	7.02 ± 0.62	0.519
SEI (Nms)	5.08 ± 1.0	5.63 ± 1.64	6.12 ± 1.78	0.071	5.04 ± 1.0	5.7 ± 1.61	6.03 ± 2.23	0.158
NJ	40.23 ± 28.39	41.63 ± 17.54	42.54 ± 25.27	0.794	40.78 ± 19.57	41.5 ± 18.06	44.59 ± 25.35	0.997
ACP	0.83	0.85	0.83	0.518	0.83	0.89	0.81	0.171

**Table 6:** Age subset: hand-to-mouth.

Age	Hand-to-mouth dominant limb				Hand-to-mouth nondominant limb			
Parameter	Young	Middle	Senior	*p* value	Young	Middle	Senior	*p* value
*T* (s)	**0.89** ± **0.15**	**0.89** ± **0.16**	**1.02** ± **0.2**	**0.03**	0.9 ± 0.15	0.88 ± 0.19	0.97 ± 0.2	0.285
SE (°)	36.86 ± 12.65	35.13 ± 14.89	34.98 ± 12.84	0.721	38.12 ± 16.08	39.9 ± 11.64	37.49 ± 13.16	0.685
EF (°)	137.31 ± 7.91	139.97 ± 11.81	142.47 ± 9.47	0.137	136.64 ± 8.25	137.48 ± 9.91	139.24 ± 10.32	0.443
ST (Nm)	4.05 ± 1.03	4.5 ± 0.78	4.3 ± 0.99	0.385	4.18 ± 1.03	4.5 ± 1.14	4.76 ± 1.16	0.08
SEI (Nms)	**3.12** ± **0.99**	**3.24** ± **0.85**	**3.73** ± **1.09**	**0.045**	3.29 ± 1.18	3.5 ± 0.99	3.9 ± 1.35	0.216
NJ	**18.94** ± **9.02**	**20.49** ± **8.59**	**27.28** ± **13.72**	**0.024**	24.51 ± 28.31	18.55 ± 7.37	23.92 ± 10.33	0.231
ACP	0.84	0.87	0.8	0.218	0.84	0.89	0.83	0.121

**Table 7:** (*T*, motor control indexes) regression—reaching movement.

RM							
Normalized jerk							
Dominant				Nondominant			
Young adults	Middle aged	Senior	All	Young adults	Middle aged	Senior	All

*R* = 0.49 *p* = 0.004	*R* = 0.81 *p* < 10^–4^	*R* = 0.62 *p* = 0.002	*R* = 0.64 *p* < 10^–9^	*R* = 0.48 *p* = 0.004	*R* = 0.40 *p* = 0.088	*R* = 0.69 *p* < 10^–3^	*R* = 0.59 *p* < 10^–7^

Acceleration coefficient of periodicity							
Dominant				Nondominant			
Young adults	Middle aged	Senior	All	Young adults	Middle aged	Senior	All

*R* = −0.20 *p* = 0.26	*R* = −0.63 *p* = 0.004	*R* = −0.67 *p* < 10^–3^	*R* = −0.49 *p* < 10^–5^	*R* = −0.25 *p* = 0.15	*R* = −0.87 *p* < 10^–5^	*R* = −0.73 *p* < 10^–5^	*R* = 0.59 *p* < 10^–7^

**Table 8:** (*T*, motor control indexes) regression—hand-to-mouth movement.

HtMM							
Normalized jerk							
Dominant				Nondominant			
Young adults	Middle aged	Senior	All	Young adults	Middle aged	Senior	All

*R* = 0.75 *p* < 10^–6^	*R* = 0.84 *p* < 10^–5^	*R* = 0.79 *p* < 10^–5^	*R* = 0.80 *p* < 10^–17^	*R* = 0.75 *p* < 10^–6^	*R* = 0.65 *p* = 0.002	*R* = 0.67 *p* < 10^–3^	*R* = 0.71 *p* < 10^–12^

Acceleration coefficient of periodicity							
Dominant				Nondominant			
Young adults	Middle aged	Senior	All	Young adults	Middle aged	Senior	All

*R* = −0.43 *p* = 0.010	*R* = −0.63 *p* = 0.004	*R* = −0.66 *p* < 10^–3^	*R* = −0.61 *p* < 10^–8^	*R* = −0.61 *p* < 10^–3^	*R* = −0.36 *p* = 0.135	*R* = −0.47 *p* = 0.022	*R* = −0.51 *p* < 10^–5^

## Abbreviations

FHfFC: Future Home for Future Communities
RM: Reaching movement
HtMM: Hand-to-mouth movement
ICF: International Classification of Functioning
M: Males
F: Females
T: Movement time (forward phase)
SE: Shoulder elevation
EF: Elbow flexion
ST: Shoulder torque
SEI: Shoulder effort index
NJ: Normalized jerk
ACP: Acceleration coefficient of periodicity.

## Additional Points

*Consent for Publication*. The authors gave their consent for publication. *Availability of Data and Material*. The data sets used and/or analyzed in the current study is available from the corresponding author on reasonable request.
